# Alien species pathways to the Galapagos Islands, Ecuador

**DOI:** 10.1371/journal.pone.0184379

**Published:** 2017-09-13

**Authors:** M. Verónica Toral-Granda, Charlotte E. Causton, Heinke Jäger, Mandy Trueman, Juan Carlos Izurieta, Eddy Araujo, Marilyn Cruz, Kerstin K. Zander, Arturo Izurieta, Stephen T. Garnett

**Affiliations:** 1 Research Institute for the Environment and Livelihoods, Charles Darwin University, Casuarina, Northern Territory, Australia; 2 Charles Darwin Foundation, Puerto Ayora, Galápagos Islands, Ecuador; 3 Ministerio de Turismo del Ecuador-Observatorio de Turismo de Galápagos, Puerto Ayora, Galápagos Islands, Ecuador; 4 Dirección del Parque Nacional Galápagos, Puerto Ayora, Galápagos Islands, Ecuador; 5 Agencia de Bioseguridad de Galápagos, Puerto Ayora, Galápagos Islands, Ecuador; 6 Northern Institute, Charles Darwin University, Casuarina, Northern Territory, Australia; Chinese Academy of Agricultural Sciences Institute of Plant Protection, CHINA

## Abstract

Alien species, one of the biggest threats to natural ecosystems worldwide, are of particular concern for oceanic archipelagos such as Galápagos. To enable more effective management of alien species, we reviewed, collated and analysed all available records of alien species for Galápagos. We also assembled a comprehensive dataset on pathways to and among the Galápagos Islands, including tourist and resident numbers, tourist vessels, their itineraries and visitation sites, aircraft capacity and occupancy, air and sea cargo and biosecurity interceptions. So far, 1,579 alien terrestrial and marine species have been introduced to Galápagos by humans. Of these, 1,476 have become established. Almost half of these were intentional introductions, mostly of plants. Most unintentional introductions arrived on plants and plant associated material, followed by transport vehicles, and commodities (in particular fruit and vegetables). The number, frequency and geographic origin of pathways for the arrival and dispersal of alien species to and within Galápagos have increased over time, tracking closely the increase in human population (residents and tourists) on the islands. Intentional introductions of alien species should decline as biosecurity is strengthened but there is a danger that unintentional introductions will increase further as tourism on Galápagos expands. This unique world heritage site will only retain its biodiversity values if the pathways for invasion are managed effectively.

## Introduction

Alien species cause such high levels of biodiversity loss [1] and environmental change [2, 3] that they are the subject of a dedicated target for action under the Convention on Biological Diversity (Aichi target 9). This target states that ‘by 2020, invasive alien species and pathways are identified and prioritized, priority species are controlled or eradicated and measures are in place to manage pathways to prevent their introduction and establishment’ [4]. Alien species are a particularly important contributor to biodiversity loss on oceanic islands [5, 6].

A primary driver of the increasingly rapid pace of biological invasions is the accelerating rate of movement of people and goods worldwide [1, 7, 8]. While trade-related transport is the principal pathway for invasions [9], tourism is also an important enabler of intentional or unintentional movement of alien species [10–12]. This is likely to increase because tourism is becoming one of the largest and fastest growing sectors of the global economy [13].

For the isolated Galápagos Islands, alien species are the most serious biodiversity threat [14], followed by the impacts associated with a growing residential and temporary human population [15]. Galápagos has exceptionally high levels of endemism [16], which led to its recognition as a UNESCO World Heritage site, a Priority Ecoregion for Global Conservation [17], a ‘flagship’ area for conservation [18], and one of the 137 most irreplaceable protected areas in the world [19]. These same qualities have made Galápagos an important hub for nature-based tourism [20] that now underpins the entire local economy [21–23].

Although Galápagos was discovered in 1535, it was not permanently inhabited until 1832 [24]. For the 140 years of colonization before tourism began in earnest in the late 1970s [20], local residents relied on fishing and agriculture [25]. For the last 40 years tourist visitation rates have increased exponentially. This has attracted migrants from mainland Ecuador to service the industry [18] and increased the volume of cargo coming from mainland Ecuador to satisfy the needs of the growing transient and resident population [26, 27].

The first alien species (e.g. goats and rats) were introduced by whalers and buccaneers between 1685 and 1850 [28]. Introductions increased with permanent colonisation by the agricultural and fishing communities and even further once tourism supported an expanding residential population. In recognition of the risks posed by alien species, the Ecuadorian government implemented biosecurity protocols for Galápagos in 1999, including a list of permitted, restricted and prohibited produce and goods [27]. This was followed by the development of an invasive alien species management strategic plan in 2007 (Plan de Control Total de Especies Introducidas) and, in 2012, by the formation of a dedicated biosecurity agency (Agencia de Bioseguridad de Galápagos: ABG) with a mandate to prevent and reduce the risks of introduction of alien species that affect human wellbeing, biodiversity and ecosystem integrity.

One reason for continued introductions can be inadequate understanding of alien species pathways to and within Galápagos; information that is vital to prevent new incursions [8]. Previous studies have linked alien species introductions and tourism in Galápagos [29–33] but none have attempted to collate all available information on alien species pathways. Supporting the aspirations of Aichi Target 9, the aim of our study was to: 1) analyse alien species pathways to Galápagos and among its islands; 2) assess the importance of different pathways over time; and 3) provide recommendations for strengthening biosecurity.

## Materials and methods

### Study site

**Fig 1:**
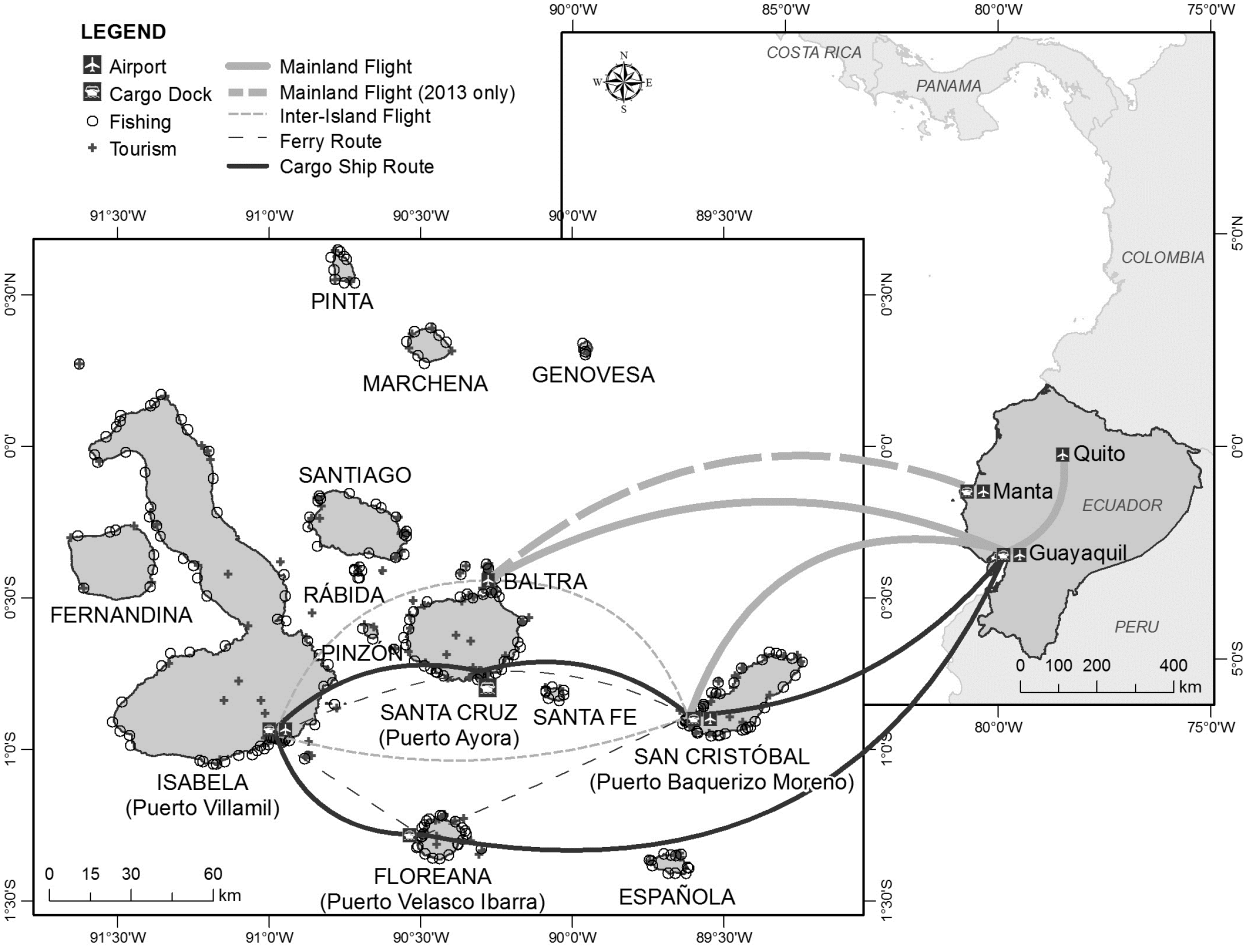
The Galápagos Islands in relation to mainland South America. The Galápagos Islands and mainland Ecuador showing air (grey lines) and sea routes (black lines), ports, tourism sites (black crosses) and fishing sites (open circles). The remote and rarely visited northernmost islands (Darwin and Wolf) are omitted for clarity of presentation.

The Galápagos Islands are a remote oceanic archipelago 1,000 km west of mainland Ecuador consisting of 13 large islands and over 100 small islands, islets and rocks [34] with a total land area of ca. 8,000 km^2^ (Fig 1). The resident population of about 25,000 is spread among just five islands. Most people (11,800) live in the commercial and economic hub of Puerto Ayora on Santa Cruz, followed by 6,500 inhabitants in the provincial capital, Puerto Baquerizo Moreno, on San Cristóbal. Puerto Villamil, on Isabela, has 2,160 and Puerto Velasco Ibarra, on Floreana, has 111 residents. The remaining population (4,600) live in small communities in the highlands of the inhabited islands. No information is available on the population of Baltra where the few residents are associated with the air force base [35]. Agricultural areas, together with urban settlements, account for less than 3% of the total terrestrial area of the islands [36]. The Galápagos Archipelago was declared a National Park in 1959 and its surrounding waters a marine reserve in 1998 [36].

### Source databases

To understand the pathways, or means by which species are transported from one location to another [9, 37], we assembled data on alien species recorded in Galápagos, human population trends, the transport of people and their cargo, and the frequency of visitation to sites on the islands. Data relevant to pathways were retrieved from six institutions (Table 1) and organized under three main topics: human population, transport and alien species.

**Table 1:** Data sources.

Source	Information	Data gathering methodology	Missing information	Geographical area	Years
Galápagos National Park Directorate (Dirección del Parque Nacional Galápagos—DPNG)	Number of visitors	Data was largely derived from questionnaires that all visitors to the islands are obliged to complete upon arrival to the islands. Coverage is therefore likely to be close to 100%.	Number of transient workers	Galápagos Islands	1979–2015
	Number of tourism vessels	Database of licenses issued annually. Includes number and characteristics of the tourist vessels. Covers 100% of legal tourist operations.		Galápagos National Park and Marine Reserve	2014
	Itinerary of private yachts	Database of permits issued by DPNG.	Last port of call	Galápagos National Park and Marine Reserve	2014
	Number of tourist sites	Information from DPNG published in De Groot [39], [40], and DPNG [36].		Galápagos Islands	1983, 1996, 2007, 2014
General Directorate of Civil Aviation	Number of flights and passengers arriving in Galápagos from mainland Ecuador	In-situ counts of plane occupancy and plane capacity registrars		Baltra airport	2010–2015
				San Cristóbal airport	2010–2015
	Air cargo (t)	Cargo registrars	San Cristóbal airport	Baltra airport	2002–2012
			Type of products	San Cristóbal and Baltra airports	2013–2014
Ecuadorian Ministry of Transport and Public Works	Interisland public transport vessels	Annual registrar		Inhabited ports	2013–2016
Ecuadorian Census Bureau	Resident population size	Population census data is gathered by teams of volunteers that visit each Galápagos household on one single day. A national census has been carried out in 1950, 1962, 1974, 1982, 1990, 2001 and 2010 by the Ecuadorian Census Bureau. During the entire month of November 2015, the Galápagos Government Council did a Galápagos-only census.		Galápagos province	1950, 1962, 1974, 1982, 1990, 2001, 2010, 2015
Agencia de Bioseguridad de Galápagos (ABG)	Sea Cargo (t)		Cargo shipped from other docks in mainland Ecuador	Cargo received in the StoreOcean dock in Guayaquil	2011–2014
	Invertebrates intercepted on cargo	Identifications of pests of biosecurity importance intercepted. Only taxa that are of biosecurity importance or are living are identified and listed. Typically these are only listed once, even if they are introduced on a regular basis.		Quito airport	2012–2016
	Products confiscated in 2015 and 2016 from passengers	Routine biosecurity inspections of passengers traveling on commercial flights from mainland Ecuador, from private, international yachts, and interisland ferries and planes.		Quito and Guayaquil airports	2015–2016
				Galápagos airports	
				Passenger and cargo docks in the Galápagos Island.	
	Biosecurity interceptions	ABG technicians carry out spot inspections of plane cabins and holds and boat cargo storage areas using insect vacuums and visual observation techniques. Technicians are unable to process and count all specimens and only search for species on their watch list.		Quito, Guayaquil and Baltra air and sea cargo depot	2013–2016
	Biosecurity retentions	Products retained by ABG biosecurity inspectors upon visual examination, X-ray revision and general biosecurity inspections. The products retained are either prohibited (http://bioseguridadGalapagos.gob.ec/lista-de-productos-final) or do not meet biosecurity standards		Quito, Guayaquil and Baltra air and sea cargo depot	2015–2016
	Number of private yachts	From quarantine and biosecurity inspections upon arrival to Galápagos.		Inhabited ports	2013–2015
Charles Darwin Foundation	Number of alien species	Most recent numbers for non-native species were retrieved from the database of the Charles Darwin Foundation (CDF) [41]. Data was collected during surveys by CDF, DPNG and ABG personnel in both inhabited and uninhabited islands and also includes contributions from visiting scientists and the public in general.		Galápagos	1600–2017

Human population: People were counted as either permanent residents (1833 to 2015) or tourists (1979 to 2015) (Table 1). Tourists were characterised by country of origin and as either live-aboard—those on package tours visiting islands while living aboard a boat, or land-based—tourists accommodated in a town and taking day trips alone or in groups to tourist sites within the protected area or to other human settlements. No information was available for transient workers (“*Transeúntes*”).

Transport: Data were assembled on the number and type of boats and planes used to move people and cargo to and within the archipelago, the routes taken, the frequency with which different routes were travelled, the number of passengers and the volume of cargo.

Alien species: Data were assembled on alien species (species introduced by humans beyond their native range [8, 38]) recorded in Galápagos, including species that have become established, those intercepted or eradicated after introduction, and those only known from historical records. The sources for these data were the Charles Darwin Foundation Introduced Species Database and the ABG (Table 1). Terrestrial plants include varieties and cultivars; vertebrates include terrestrial and freshwater species. The pathway of introduction for each alien species was determined by CEC, HJ, and other Galápagos invasive species experts using the framework devised by Hulme [9] and adopted by the Convention of Biological Diversity [37]. Minor adaptations were made to the framework to better fit the situation in Galápagos including replacing and combining the main pathways “Escape” and “Release” with “Intentional”. This allowed separation of the pathway from the current status of the species in the archipelago, which has the potential to change. Furthermore, the definition for the main pathway “contaminant” was altered to consider the unintentional movement of alien species on live organisms or other material that are not commodities (items of use or value) and may have been introduced unintentionally (Table 2, S1 Table). Minor changes were made to the text of some subpathways for clarification (Table 2, S1 Table) and an additional pathway “Hitchhiker on transport vehicles/cargo” was included for alien species that were known to have come in on either boats or planes, but may have been associated with the cargo rather than the transport vehicle *per se*. Stowaways intercepted in cargo or personal luggage were assigned to the same group because the data did not allow us to distinguish between the two pathways. Alien species were assigned to one of seven categories that best reflect their current status in Galápagos (Table 2, S1 Table). The ABG provided additional data on alien species interceptions including products confiscated by biosecurity inspectors that were not on the list of products approved for import to the Galápagos Islands (http://bioseguridadGalapagos.gob.ec/lista-de-productos-final) or that did not meet the ABG import requirements (Table 1). The ABG also provided records of species considered a biosecurity risk that had been intercepted in the cabins and holds of planes arriving in Galápagos.

**Table 2:** Pathways of introduction and current status of alien species recorded in the Galápagos Islands.

			Marine invertebrates	Marine plants	Pathogens	Terrestrial Insects	Terrestrial Invertebrates	Terrestrial Plants	Vertebrates	Total
**Total number of recorded alien species**			**21**	**2**	**63**	**545**	**77**	**821**	**50**	**1579**
**Pathway of introduction**	**Intentional**	Agriculture/Horticulture						687		687
		Biocontrol				1				1
		Animals for breeding					1		16	17
		Fishery in the wild							2	2
		Food with potential to propagate	1					4		5
		Pet/aquarium/terrarium species							11	11
		Release in nature for use							1	1
	**Unintentional: Contaminant**	Contaminants of plants (inc. seeds and plant associated material				196	11	127		334
		Food contaminant				89	2			91
		Parasites/pathogens on animals			37	35	4			76
		Parasites/pathogens on plants			26					26
		On habitat material (soil, vegetation)				95	35			130
		Wood/Construction material				13				13
	**Unintentional: Stowaway**	Hitchhiker on airplanes				3	2		1	6
		Hitchhiker on boats				16	2		11	29
		Hitchhiker on transport vehicles/cargo				78	15		6	99
		Ship hull	18							18
		Unknown	1	2						3
	**Unknown**	Unknown	1			19	5	3	2	30
**Total number of established alien species**			**5**	**2**	**63**	**499**	**70**	**810**	**27**	**1476**
**Status in Galápagos**	**Established**	Naturalized	5	2	38	467	68	270	18	868
		Present but status unknown			8	10		6	1	25
		Coexist with introduced species			17	15	2			34
		Human dependent				7		534	8	549
	**Absent**	Eradicated						2	2	4
		Historical record	1			8			8	17
		Intercepted	15			38	7	9	13	82
**% alien species introduced post 1970s**			86%	0%	100%	72%	79%	78%	52%	76%

The main pathways are: a) Intentional—live taxa deliberately brought in by humans for use; b) Unintentional: Contaminant—live taxa brought in accidentally on commodities or associated material or as contaminants of live taxa and other material that has been introduced unintentionally; c) Unintentional: Stowaway—introduction of live organisms attached to or associated with transport vessels (and associated cargo), or with personal luggage; d) Unintentional: Unknown—no data available. The status of all extant alien species in Galápagos was categorized as: a) Naturalized—reproduces and spreads in the wild without human intervention [48]; b) Human dependent—can only reproduce with human help and/or is restricted to human settlements; c) Coexists with alien species—exclusively associated with introduced species. Not necessarily restricted to human settlements: d) Historic record—organism known only from publications with no current record; e) Eradicated—organism eliminated from archipelago through deliberate intervention; f) Intercepted—organism seized in biosecurity procedures and destroyed or returned to mainland; and (g) Unknown.

### Analysis

Total human population size in each census year, calculated as the total resident population and the average number of tourists per day (number of tourists p.a. divided by 365 and multiplied by average stay length in days) was correlated with the number of recorded alien species using Pearson’s correlation coefficient. For each kingdom/class, the rate of alien species introduction was summarized by decade and totals calculated by pathway of introduction and current status in Galápagos.

## Results

### Population (1950–2015)

The resident population in Galápagos has grown from 120 inhabitants in 1833 [42] to 25,244 inhabitants in 2015 [35]. Spurts in population growth between 1950 and 1974 were associated with an earthquake in Ecuador's Tungurahua Province in August 1949 and a drought in the Loja Province during the 1960s. Tourism started in earnest in the late 1970s. The rate of increase in the resident population grew from 4.0% p.a. in 1984 to 6.4% p.a. in 2001 and later decreased to 3.1% p.a. by 2010 and 1.8% p.a. by 2015 [35].

The total number of tourists arriving each year has increased from 11,765 in 1979 to 224,755 tourists in 2015, a 19-fold increase. Between 2000 and 2015, average tourism growth was 8.2% (± 3.14 standard error of the mean—SEM) p.a. The average number of tourists per day has increased from 328 tourists/day in 1982 to 3,324 tourists/day in 2010 and and 4,310 tourists/day in 2015. Since 2009, there has been a rapid increase in the number of tourists choosing to stay in land-based accommodations and in 2010, there were, for the first time, more tourists staying on land (54%) than on live-aboard boats (46%). The proportion of tourists staying on live-aboard boats has steadily decreased since then to 32% in 2015. In 2015, the most common length of stay for on-board tourists was seven days, whilst land-based tourists stayed between three and four days. The number of nationalities represented among tourists increased from 93 in 2000 to 158 countries in 2014. Most tourists were from Ecuador (33.5%) followed by the USA (27.5%), United Kingdom (6.6%), Germany (4.4%) and Canada (3.7%). Tourists from other countries accounted for about 25% of the total. In 2015, combining residents and tourists (and excluding transient workers, for whom data were not available), there were approximately 30,000 people present in Galápagos on any given day.

### Transport routes and destinations

#### Air travel (2010–2015)

**Fig 2:**
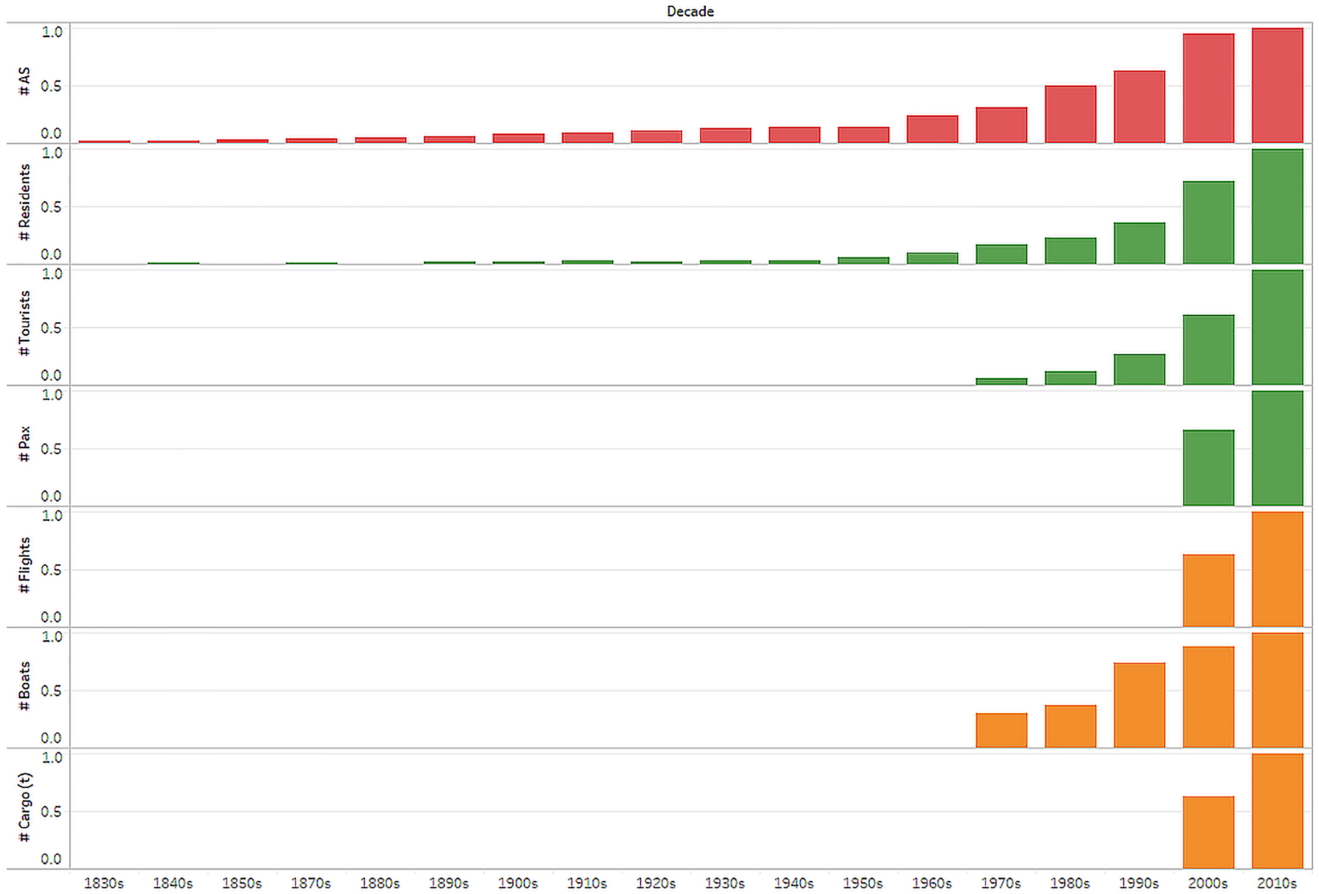
Number of alien species (AS) and number of vectors. Normalised sum of alien species (AS), residents, tourists (national and foreign), plane passengers (PAX), flights, boats (tourist+cargo) and, cargo per decade in the Galápagos Islands, Ecuador.

Most flights bound for Galápagos originated in Quito with a stopover in Guayaquil to pick up additional passengers and/or cargo (Fig 1). From 2010 to 2015, most passengers flew to Baltra Island (160,000–215,000 passengers/year), some went to San Cristóbal Island (50,000–78,000 passengers/year) and at least one flight per year flew directly from Manta to Galápagos in 2012 and 2013. The number of commercial flights to Galápagos increased from 3,854 in 2010 to 5,566 in 2015, which represents an increase from 74 flights a week in 2010 to 107 in 2015. Flights per week to Baltra have increased from 54 in 2010 to 78 in 2015, whilst there were 21 flights per week to San Cristóbal in 2010 and 29 in 2015 (Fig 2, S1 Fig) Plane seat occupancy for all flights to Galápagos was 68–76%, with some passenger payload being taken up by cargo. Of passengers transported to and from Baltra airport, the airport with the highest visitation rates, 39–41% were tourists and the remainder were Galápagos Island residents or transient workers.

An average of 2,700 tonnes of cargo (± 234 SEM) p.a. were transported from mainland Ecuador to Baltra between 2002 and 2012. Long-term data was not available for San Cristóbal. Tonnage of cargo airshipped on commercial flights to Baltra and San Cristóbal airports in 2013 and 2014 was ca. 3,900 tonnes and 1,600 tonnes p.a., respectively. Cargo planes periodically travel from Guayaquil to Baltra and military planes travel from Guayaquil to Isabela island, but no data were available on frequency.

Between 2010 and 2015, a total of 1,165 private planes visited Galápagos from outside the archipelago (average of 195 ± 28 SEM p.a.). Most landed in Baltra (52%), followed by San Cristóbal (34%) and Isabela (12%). Information regarding the last port of call of these planes or the number of passengers transported was unavailable.

Regular interisland flights connect the three local airports on Baltra, Isabela and San Cristóbal Islands. More flights and passengers landed in Isabela (6,800 flights; ca. 35,000 passengers) than San Cristóbal (6,000 flights; ca. 22,000 passengers) or Baltra (5,200 flights; ca. 22,870 passengers). There is some overlap in these figures as some flights and passengers visited multiple islands.

#### Maritime travel (2010–2015)

Up to five cargo boats/month traveled from Guayaquil to Galápagos between 2010 and 2015. Before 2015, cargo boats docked at San Cristóbal first and then traveled to Santa Cruz, from where cargo was taken to Isabela and Floreana by small boats. In 2016, the government initiated a direct cargo boat route from Guayaquil to Floreana and Isabela. Since 2015, all cargo shipped to Galápagos has been initially stored in containers at containment facilities at the Caraguay and/or StoreOcean docks in Guayaquil and since 2016, at the Puertogal dock (also in Guayaquil). Information was only available for cargo shipped through the StoreOcean dock. Between 2012 and 2014, an average of 65,000 t of cargo p.a. was shipped to Galápagos from this dock. About 60% of this cargo was construction materials, followed by dry food and grains (20% each), and fresh produce and miscellaneous goods (10% each).

A total of 635 private yachts visited the islands between 2013 and 2015 with 270 in 2015 alone. Private yachts that visit Galápagos are able to travel around the islands visiting the same designated tourist sites used by tourist boats operating within the islands. No information of last port of call or number of passengers was available.

The number of tourism live-aboard vessels increased from about 40 vessels with 597 berths in 1981 to 74 vessels with 1,740 berths in 2015. No historic information was available for day-tour operations but, in 2015, there were 82 day-tour boats capable of carrying a total of 914 passengers per day. Most of the government approved tourist vessels depart from Santa Cruz Island (98 boats—live-aboard and day-tour modality), with smaller numbers from San Cristóbal, Floreana, Isabela and Baltra Islands.

In 2015, for passengers (tourists and residents) wishing to travel among the inhabited islands, there were 38 boats registered with a capacity to ferry 857 passengers/day; most of these boats are based on Santa Cruz Island. In 2015, a total of 252,104 passengers travelled among the inhabited islands with most departing from Santa Cruz (126,729 passengers), followed by Isabela (73,106 passengers), San Cristóbal (44,061 passengers) and Floreana (8,208 passengers), which represents an average of 690 passengers transported per day.

In addition, 416 artisanal fishing vessels were registered in Galápagos in 2015 [43] of which an unknown proportion is travelling within the archipelago, depending on the fishing season and resource availability. Information on routes taken by the fishing boats was unavailable. In 2015, the Galápagos National Park Directorate (Dirección del Parque Nacional Galápagos: DPNG) had 16 patrol vessels boats and one stationary pontoon, out of which only one was fully operational and seven had limited operational capacity [44], but no information was available on routes taken. Information on the number of patrol vessels operated by the Ecuadorian Navy was unavailable.

In 2015, there was a total of at least 892 boats navigating within the Galápagos Marine Reserve. On any given day of 2015, assuming that all registered tourist and passenger vessels were at maximum capacity, there were ca. 3,500 people traveling on tourist boats or ferries around Galápagos. There were 169 land and marine sites in protected areas available for tourist visits in 2014 [36], compared to 35 terrestrial sites in 1983 [39]. Additionally, there are 320 fishing sites distributed around the coastal perimeter of the islands (Jorge Ramírez, pers. comm., S1 File) (Fig 1).

### Alien species

Up to 2017, a total of 1,579 alien species are recorded as intentional or unintentionally introduced to Galápagos since their discovery in 1535, representing ca. three species per year. This includes 82 species that were intercepted, 17 that are historical records only (species may not have established or reports were erroneous) and four species that have been eradicated under management programs (rock pigeon, kudzu, tilapia and an *Opuntia* species; Table 2, S1 Table). Two species of blackberry, previously reported as erradicated [45], have been sighted recently and their current status is unclear.

About 46% of the recorded alien species were brought intentionally by humans; mostly plants, but also vertebrates (mammals (17 species), birds (10 species), reptiles (1 species) and fishes (2 species)), an insect and a terrestrial invertebrate. Humans were responsible for intentionally transporting 60% of the recorded alien vertebrates and 84% of terrestrial alien plants to Galápagos (Table 2, S1 Table). Just over half of the alien species recorded in Galápagos were introduced unintentionally through human activities, predominantly insects, followed by plants (Table 2, S1 Table). The mode of introduction for 30 species could not be determined.

Plant species intentionally introduced for agriculture/horticulture account for the most important intentional pathway of introduction (Table 2, S1 Table). The most common pathway for species introduced unintentionally was as a contaminant of plants (including seeds and plant associated material). In addition, 26 alien pathogens were probably introduced on plants. Planes and boats were the second most important pathway for unintentional introductions of animals (nine reptile species, one bird, four mammals, four amphibians, 97 insects and 19 other terrestrial invertebrates; Table 2, S1 Table). However, in 65% of the cases it was hard to determine whether the pathway was the transport vehicle itself or whether the alien species stowed away on the cargo that the transport vehicle was carrying. As for marine species, it is certain that at least 18 species arrived on ship hulls. Soil or decomposing vegetation (including animal dung) and food commodities were the third and fourth most common pathways facilitating the introduction of terrestrial insects and invertebrates. Alien vertebrates were pathways for at least 37 alien microrganisms (nematodes, bacteria, virus and protozoa), 11 insects and four arachnids. In addition, 19 hymenopteran parasitoids and three entomopathogens have been introduced to Galápagos, most of which were probably introduced via their insect hosts.

**Fig 3:**
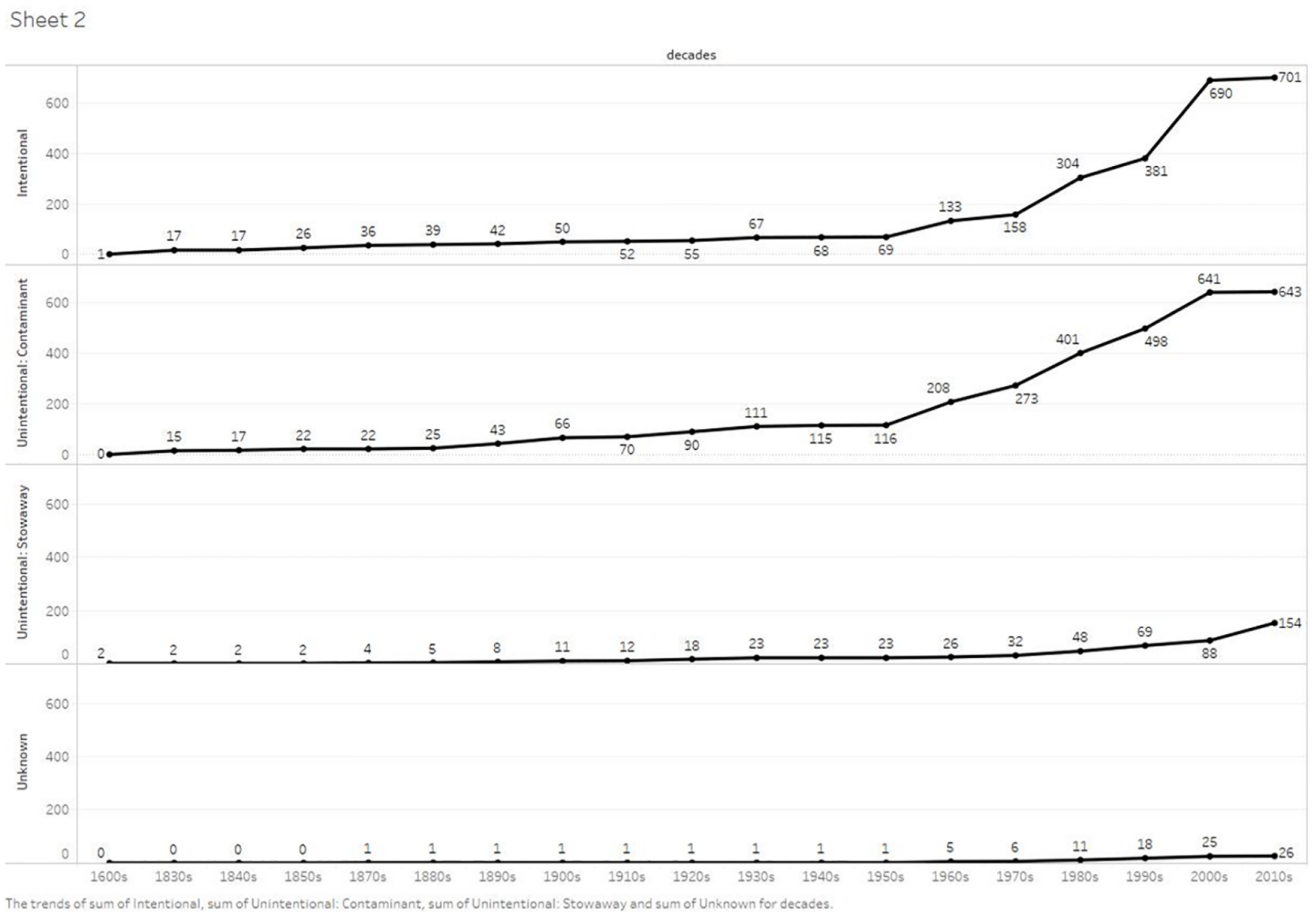
Accumulated number of alien species introduced by different pathways per decade.

**Fig 4:**
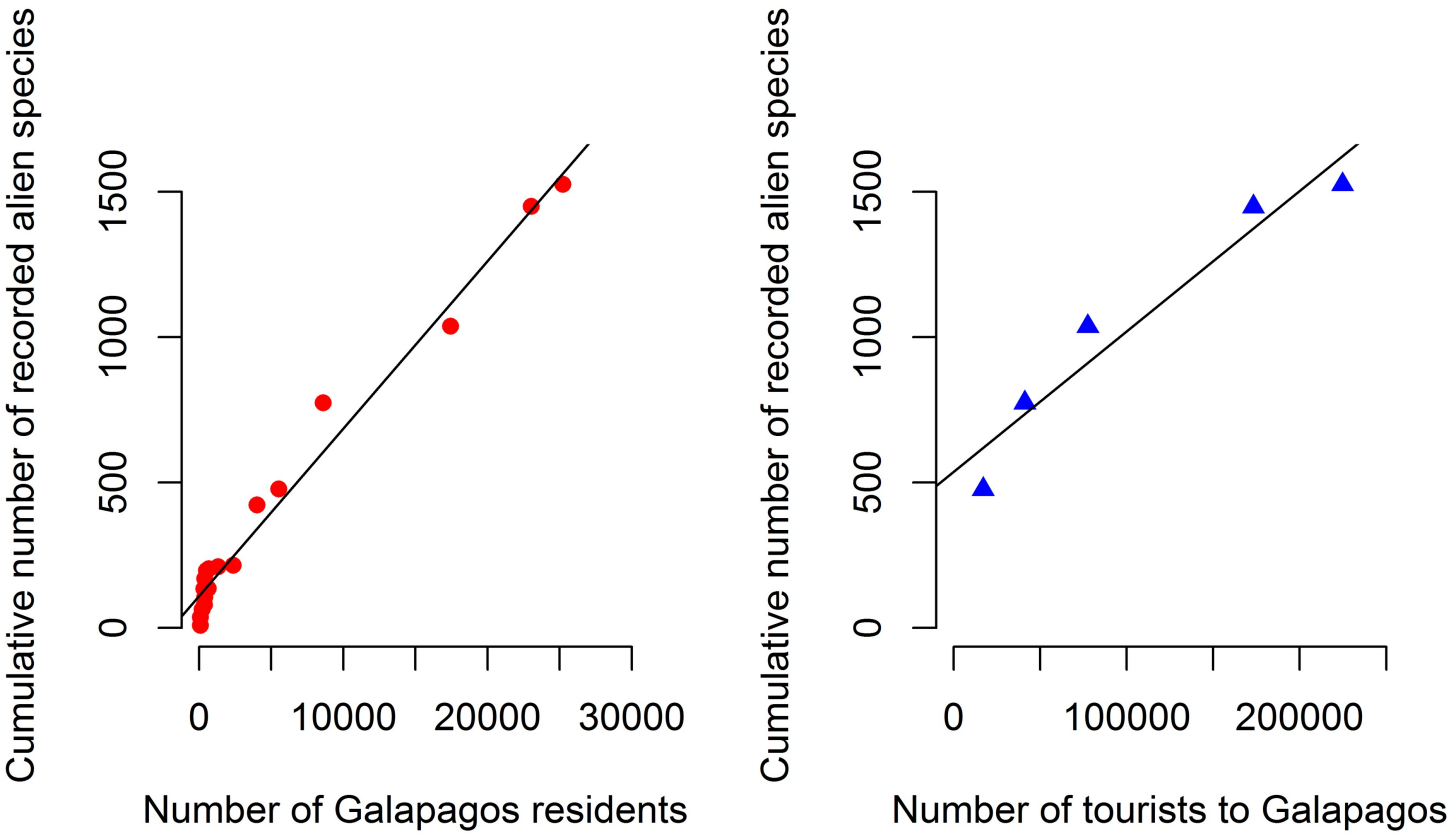
Correlation of the cumulative number of recorded alien species versus the number of Galápagos residents from 1833–2015 (R^2^ = 0.97) (red circles) and number of tourists that have arrived to Galápagos 1979–2015 (R^2^ = 0.93) (blue triangles).

Most alien species were first recorded in Galápagos within the last 50 years, with 76% of the intentional and unintentional introductions registered after 1970 (Table 2 and S1 Table, Fig 3 and S2 Fig), an average of 27 species per year for the past 40 years. The number of alien species known to be present in Galápagos was positively and closely correlated with both the total number of residents from 1833–2015 (R^2^ = 0.97) and the number of tourists who visited 1979–2015 (R^2^ = 0.93) (Fig 4, S3 Fig).

Data collected by the ABG indicates that both intentional and unintentional introductions of alien species are continuing. In 2015 and 2016, the ABG confiscated 14,180 products during routine inspections of passengers, luggage and cargo at air and sea ports in mainland Ecuador and Galápagos. Of these, 48% were products that are prohibited from entering Galápagos because they pose a threat themselves or are vectors of alien species, 36% were restricted (did not meet specific quarantine and biosecurity requirements), 11% were in poor condition and 5% were infested with pests. No information about the type of products that were confiscated or the pests associated with these products was available for 2015; however, data for 2016 showed that numerous attempts were made to introduce fruits, seeds or plant species that are prohibited from entering Galápagos, including nine species that are not currently registered as being present. Of people carrying non-permitted produce in 2015 and 2016 combined, 69% were tourists (Ecuadorian and foreign) and 31% were Galápagos residents.

Data on interceptions made during inspections of personal luggage or cargo at Quito and Guayaquil airports provide insight into the wide array of taxon groups that potentially could be transported to Galápagos. Of thousands of samples collected during routine inspections for invertebrates in the holds and cabins of planes that arrived in the Galápagos Islands from 2012 to 2016, 142 species were singled out either because they posed potential biosecurity risks and/or were living. Of these, 39 terrestrial invertebrate species would have been new records for Galápagos. Species of high biosecurity risk included two *Linepithema* species, one of which, *Linepithema humile*, is the highly invasive Argentine ant. Other notable species included leafcutting ants (*Azteca* spp.), *Apis mellifera*, a *Bactrocera* fruit fly (Tephritidae) and live ticks (*Amblyomma* sp.). The frequency of introduction/interception of these and other species of biosecurity risk was not registered. In 2016, 411 terrestrial invertebrates belonging to 14 different orders were intercepted. Lepidoptera, mainly associated with Brassicaceae, were the most common order intercepted (112 species), followed by slugs (Stylommatophora; 52 species), aphids and mealybugs (Hemiptera; 46 species). Most of the invertebrates were intercepted on fresh produce that was being transported as cargo to Galápagos.

An increasing variety of vertebrates have also been intercepted in the last few years: most are believed to have come in on transport vehicles. Citizen reports led to interception of stowaways such as an opossum (*Didelphis marsupialis*), a Sinaloan milk snake (*Lampropeltis triangulum sinaloae*), green iguanas (*Iguana iguana*) and a boa (*Boa constrictor*). In 2014, a Saffron finch (*Sicalis flaveola*) was recorded for the first time on a commercial plane. Rock pigeons (*Columba livia*), eradicated from Galápagos in the 2000s [46], were detected again in San Cristóbal in 2015. Some vertebrates have been deliberately introduced in the last decade including Tilapia (*Oreochromis niloticus*) around 2005, new dog breeds and goldfish (*Carassius auratus*) in 2016.

At least 1,476 alien species have become established in Galápagos, most of which are terrestrial plants or insects (Table 2, S1 Table). Of these species, at least 59% have become naturalized in the Galápagos National Park and have self-sustaining populations; mostly insects followed by terrestrial plants and non-insect terrestrial invertebrates (Table 2, S1 Table). Most of the pathogens that have naturalized are plant associated, although there are three viruses that have been found in both introduced and native bird species [47]. About 37% of species established in Galápagos are either dependent on humans and/or are limited to areas with human settlements and do not appear to have spread to National Park areas. These are mainly terrestrial plants, but also include vertebrates and insect species. An additional 2% of the species that are established are thought to be exclusively associated with introduced species. The status of ca. 2% of the species is unknown (Table 2, S1 Table).

## Discussion

### Alien species pathways

Over the 481 years since Galápagos was first visited by people, at least 1,579 species not previously present have been introduced by humans on at least one occasion. For the first 440 years (1535–1975), they arrived at an average of less than one species per year. Since tourism accelerated in the 1970s [20], nearly 30 new alien species have been recorded annually. As in other countries [49], a positive and significant correlation between the prevalence and density of alien species and the Human Development Index (HDI) may in part be explained by increased inventory efforts for alien species. This is particularily so for alien plant species in Galápagos; thorough botanical surveys have only been carried out in the last 30 years [50]. Furthermore, before this, cultivated species were not included in the list of alien species [50, 51]. Similarly, systematic archipelago-wide insect surveys were only initiated in the 1980s and human settlements were only intensively surveyed for insects in the 2000s [52–55]. Notwithstanding, there is a strong relationship between the number of recorded alien species and the number of residents and tourists visiting the islands.

Almost half of the alien species were intentionally introduced by humans. Some of the drivers of these intentional introductions include expanding human settlements, a desire to conduct life styles similar to those on the mainland [56] and a general lack of awareness of the possible consequences of the introduction of alien species. Most of these introductions were plant species that were introduced for agriculture or as ornamentals, but also include animals introduced for livestock (e.g. cattle, poultry, guinea pigs) or as pets (e.g. dogs, cats). Only two terrestrial invertebrate species have been introduced intentionally. One was the sanctioned introduction of a biological control agent *Rodolia cardinalis* [57]. In the other case, the Giant African Land Snail (*Lissachatina fulica*) was introduced with the intent of breeding it in captivity to produce a face cream with purported regenerative properties. It is also possible that one marine species, the mouthless crab–*Cardisoma crassum*, was introduced for human consumption and escaped into the wild [58].

Fewer vertebrates have been introduced deliberately since the implementation of biosecurity protocols, but they continue to arrive. Gravid females and carriers of pathogens are likely to pose the highest risks to Galápagos biota, especially species that are closely related to native fauna, e.g. finches [59] and iguanas [60]. Some domestic species, such as dogs, cats and domestic fowl, have arrived with pathogens [61], some of which have transferred to native fauna [47, 62]. Research is ongoing to better understand what pathogens have been introduced to Galápagos, their effects on native wildlife and potential threats [63].

Plants and their by-products continue to be introduced on a regular basis, often because people are not fully aware of the list of permitted products. Although fewer products are being confiscated from Galápagos residents than was recorded by [27], some prohibited products are still commonly introduced by local residents and national and international tourists. This is especially true of *Passiflora* species such as granadilla, *Passiflora ligularis* (intercepted 461 times in 2016), and the banana passionfruit, *Passiflora tripartita* var. *mollissima*, also known as *P*. *mollissima* or *P*. *tarminiana* (intercepted 18 times in 2016). Granadilla has escaped cultivation and has already naturalized in Galápagos, but to our knowledge banana passionfruit is not being cultivated. The latter is invasive in Hawaii and New Zealand [64].

Plants, as is the case on other islands and at the global level [6, 65], were a primary pathway for alien species, facilitating the introduction of more than a third of the species that were introduced unintentionally including insects, other plants, terrestrial invertebrates, and pathogens. In Galápagos, many of the plant species were imported before establishment of biosecurity inspection protocols in 1999 and many were brought in as cuttings or seedlings from people’s gardens, bringing with them a large array of associated insects and pathogens [54]. Organic matter or soil that is often associated with these plants was the third most important pathway for the unintentional introduction of alien species to Galápagos, followed by contaminants on food and construction materials. Transport vehicles (boats and planes traveling to Galápagos) and their associated cargo and luggage, have been a major pathway for undetected entry of insects and other terrestrial invertebrates. Biosecurity protocols for fumigating planes and boats were implemented in 2008 [27] and ABG technicians collect large numbers of dead specimens in the holds of planes. However, recent data from the ABG suggests that individuals, including species of biosecurity importance, still arrive alive. These could escape and become established.

Ship hulls and ballast water are pathways for marine alien species world-wide [66]. Alien marine species account for only 23 of the alien species recorded as introduced to Galápagos and most are recent interceptions; however, this may be because there has been less research and survey work [58]. Additional studies are needed to confirm the identification of these species and determine whether any have successfully established in Galápagos including their pathway of introduction. Marine species are well known for being difficult to control due to intrinsic properties of marine ecosystems [66, 67], highlighting the need to increase biosecurity measures to reduce the risks of introduction and establishment of new marine species.

The elusiveness of invertebrates and other small organisms increases the probability of repeated introductions. Species with high frequencies of introduction, especially those with a wide tolerance for habitat and climate types, will have a higher chance of succesful colonization [68]. Although our analysis did not take into account the rates of introduction of particular species or taxon groups, evidence suggests that some species are arriving on a regular basis. For example, the Argentinian ant (*L*. *humile*), one of the species on the list of the 100 worst invasive alien species world-wide [69], has been intercepted at Baltra airport at least five times. This species is found in high numbers in and around the Quito airport and the ABG are now treating the airport with ant baits and implementing other techniques to lower the risks of successful colonization of Galápagos by this species. Similarly, there have been repeated incursions of the southern house mosquito (*Culex quinquefasciatus*), a species first recorded in 1985 [54, 60]. This species currently does not appear to be a vector for high risk diseases in Galápagos, but elsewhere, including mainland South America, it is a known vector of West Nile Virus (WNV) and avian malaria [70, 71].

### Human mobility and connectivity

#### External pathways

The natural biogeographic isolation of Galápagos is breached on a daily basis by (i) aeroplanes transporting tourists, residents, transient workers and cargo, (ii) cargo vessels transporting goods and produce, (iii) international private yachts, (iv) patrol boats and (v) illegal boats. The data compiled in this study indicate that without exception, both introduction pathways and rates of alien species establishment have increased enormously since tourism accelerated in the 1970s. Despite original plans to restrict the number of departure and entry points to Galápagos for planes (Guayaquil, Quito, Baltra, San Cristóbal) and for boats (Guayaquil, Baltra) in order to ensure compliance with inspection and fumigation protocols [27], there is an ongoing increase in the number of docks, airports and local ports connecting Galápagos to mainland Ecuador and among the islands themselves.

Even when planes are fumigated, there have been interceptions of live insects in the holds of planes. In some cases (authors’ experience), planes flying to Galápagos had just come from other areas or other countries (e.g. Peru), increasing the diversity of species that could be transferred. Ports of sea-going vessels are primarily restricted to Guayaquil where inspections and fumigation takes place, but international private yachts arrive from international ports, most traveling directly from Panama [58], although they are inspected upon arrival in Galápagos and records of biosecurity and quarantine inspections in their last port of call are checked.

Ecuadorian and international tourists are responsible for at least 69% of the products confiscated by biosecurity inspectors, making tourism a high risk pathway. An evaluation of the importance of personal luggage as a pathway for stowaways and contaminants was not possible with the data available but would assist in managing this risk. The shift in tourist visits from long-term on-board boat trips to short-stay land-based trips means not only an increase in tourist numbers, but also in the frequency with which people move between mainland Ecuador and the islands, thus increasing the probability of alien species incursions [72]. With tourists now coming from 81% of all countries in the world, the size of the pool of potential invaders is likely to have expanded [38]. Tourists regularly spread alien species beyond their natural range [10], mostly accidentally on their clothing, on transport vehicles or in luggage [11, 12]. Currently, the lack of quantitative information on the importance of luggage as a pathway hinders a thorough evaluation of the risk of this pathway. Scientists and their support personnel have also been shown to be responsible for introductions of alien species to remote areas [73]. This emphasises the need to evaluate and monitor all human movements around Galápagos, taking into account the geographical origin of the visitors and residents.

#### Internal pathways

Galápagos is at ‘an early stage of invasion’ which will undergo further spread of already present alien plants and animals thanks to the combined influence of time lags and human mediated propagule pressure [14]. Over 99% of the archipelago is now accessible in less than six hours of travel from entry points to Galápagos [74]. Contact between islands ocurs when (i) registered tourist vessels connect inhabited islands and uninhabited islands, (ii) small private and public planes and boats connect inhabited ports for both residents and tourists, (iii) fishers fishing legally near islands camp illegally on land, and when (iv) scientific and patrol vessels and (v) illegal fishing boats visit land. Almost all this contact originates from the inhabited towns where most alien species already present in Galápagos occur [75]. The risk of spread of alien species resulting from these contacts is increasing rapidly. By 2015, over 225,000 tourists moved within the archipelago to over 169 sites. The risk of transfer of alien species to new, unexposed sites and distant, more pristine sites is being exacerbated by an increase in the number of designated tourist sites, overlapping of sites for different types of tourist activities, and opening up of some previously restricted sites for day tours.

### Policy implications and solutions

According to the ABG data and predictions [76], the Galápagos Islands have not yet reached a saturation point for the introduction of new species. This especially applies to invertebrates of which at least 300 new alien species have been registered since 1998 [41, 54]. This will be the most challenging taxon group to control in coming years.

In Galápagos, tourism is directly or indirectly responsible for the introduction of almost all alien species. The large amount of land-based accommodation available on the islands with only moderate occupancy rates, the unfilled capacity of planes, and the ambitious tourism campaign “*All you need is Ecuador*” started by the Ecuadorian Government in 2014 [77] to attract more national and international tourists all suggest that there are aspirations for tourist numbers to increase. The likelihood of the arrival and dispersal of new and existing alien species is therefore high and has the potential to threaten both the uniqueness of the islands and its economy.

In 2007, an understaffed and underfunded quarantine and biosecurity system was estimated to be intercepting one in 8,230 organisms (June 2006 to January 2007) before they arrived in Galápagos [27]. Since establishment of the ABG in 2012, additional staff have been hired and new protocols and biosecurity systems are being put in place, including marine biosecurity measures [78]. A re-evaluation of the efficacy of the biosecurity systems is necessary to determine whether species interception rates have improved. However, even following the improvements, recent data from the ABG show that there are still new species arriving in Galápagos, including some of the 100 world’s worst invasive species [69]. Still greater investment is therefore required, especially given the anticipated continued increase in tourism and greater connectivity between the inhabited zones of Galápagos and the once isolated protected areas.

Priorization invasion policy and management needs to consider not only pathways [8] but also species and sites that are highly sensitive and susceptible to invasion and of conservation importance [79]. The emphasis of policy should be on minimising *a-priori* risks to avoid expensive reactive measures to control and eradicate alien species. Investing in a strong and effective biosecurity system, and minimizing the pathways between and within the archipelagos, will help preserve Galápagos ecosystem health and local livelihoods. Relatively simple actions that could be taken relate to cargo quarantine. Both in Guayaquil and upon arrival in Galápagos, all sea cargo should be stored in a single storage area, for later distribution within the islands. This measure would ensure that cargo is inspected and quarantined appropriately and comprehensively, thus minimising risks of further introductions. Additionally, further strengthening of the existing quarantine and biosecurity protocols for all public and private transport vehicles is necessary to reduce the rates of unintentional introduction of alien species.

Secondly, given the vulnerability of Galápagos ecosystems to alien species incursions, the increased frequency of pathways and the widening geographic origin of some of these pathways, we recommend that the needs of the ABG are evaluated based on our current knowledge of pathways. To this end, a centralised database should be established where information on the number of planes and boats of all types, tourists and residents, cargo, tourist and private boats, and biosecurity interventions are gathered for periodic analysis to understand both success and failures in alien species control.

Thirdly, educational programs of both residents and tourists should be created to help lower the rates of both intentional and unintentional introductions by people. Such programs would need periodic review and renewal to ensure that they do have the desired effect and to keep those regularly travelling to and within Galápagos engaged in the messages and the need for action.

Fourthly, a risk assessment differentiating the types of human mobility (residents vs tourists) and the different types of transport vehicles and their likelihood of bringing new alien species, should be carried out. This will identify the role of current pathways/vectors of bringing new alien species to Galápagos irrespective of their intrinsic characteristics (propagule pressure, likelihood of establishment and likelihood of becoming invasive), hence providing key information for pathway management, biosecurity and quarantine.

Finally, given that new alien species introductions cannot be fully avoided and alien species already present will continue to spread, establishment of an early detection center for both terrestrial and marine species would capitalise on the capacity of visitors and residents alike to help monitor for alien species arrival and their spread (e.g. citizen science project).

## Conclusions

This study presents the first baseline information on the alien species recorded in Galápagos as well as the relevant pathways used for their arrival, information deemed essential for addressing Aichi Target 9 [79]. It also demonstrates that alien species continue to arrive in Galápagos via a multitude of pathways that are increasing in magnitude, multitude and spatial spread, thus exarcerbating the vulnerability of Galápagos to this threat. This could potentially be aggravated by climate change, particularly by the predicted increase in frequency of El Niño events in the Eastern Pacific [80]. The unique nature of Galápagos can therefore only be sustained if there is a far more concerted effort to keep additional alien species out, or keep them from spreading once they have arrived. Given that some of the information needed for our pathways analysis was either not available or unusuable, our results are likely to under-estimate the number of alien species arrivals. The principal challenge that Galápagos faces now and in the future is how to lower the rate of accidental introductions, in particular species associated with commodities or stowaways on transport vehicles, cargo, and luggage. This challenge is expected to grow with increasing human activity and the frequency and intensity of introduction pathways. By investing in a strong and effective biosecurity system and minimizing the pathways between and within the archipelago, ecosystem health and the well-being of its inhabitants can be maintained, keeping one of the best preserved oceanic archipelago intact.

## Supporting information

S1 Fig Normalised values for the cumulative number of alien species, average number of Galapagos residents, average number of tourists, number of boats (tourism, public transport, cargo), number of flights (from mailand and within the inhabited islands), number of passengers transported by plane, and number of cargo (t) via boats and planes per decade. (XLSX)

S2 Fig Cumulative number of alien species per pathway of introduction per decade. (XLSX)

S3 Fig Total number of Galapagos residents and tourists through time against cumulative number of recorded alien species in Galapagos. (XLSX)

S1 Table Pathways of introduction and current status of alien species recorded in the Galápagos Islands. (XLSX)

S1 File Authorization by Jorge Ramirez to cite information provided on number of fishing sites around Galapagos coastline. (DOCX)
