# Supplementary material for: Alien species pathways to the Galapagos Islands, Ecuador
Source: PLoS One. 2017 Sep 13;12(9):e0184379. doi: 10.1371/journal.pone.0184379 (PMC5597199; doi:10.1371/journal.pone.0184379)
Supplement: S1 File — (DOCX) [file pone.0184379.s005.docx]

To whom it may concern,

Upon request from M. Veronica Toral Granda, PhD Candidate from Charles Darwin University, I provided the below information (in bold and underlined) regarding the number of fishing sites in the Galapagos Islands.

“There were 169 land and marine sites in protected areas available for tourist visits in 2014 [36], compared to 35 terrestrial sites in 1983 [42]. **Additionally, there are now 320 fishing sites distributed around the coastal perimeter of the islands (Jorge Ramírez, pers. comm .) (Fig 1).”**

I authorise Veronica to use this information in her paper.


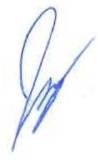


Jorge Ramírez WWF-ECUADOR

[jorge.ramirez@wwf.org.ec](mailto:jorge.ramirez@wwf.org.ec)
